# Risk Factors for Prognosis of Lung Cancer Patients Receiving Anlotinib Treatment: A Retrospective Cohort Study

**DOI:** 10.1111/crj.70051

**Published:** 2025-02-09

**Authors:** Congyi Xie, Jinzhan Chen, Shuwen Yang, Feiyang Ye, Zhenyang Lin, Yijiao Xu, Yimin Yang, Lin Tong

**Affiliations:** ^1^ Department of Pulmonary Medicine Zhongshan Hospital (Xiamen), Fudan University Xiamen Fujian People's Republic of China; ^2^ Xiamen Clinical Research Center for Cancer Therapy Xiamen Fujian People's Republic of China; ^3^ College of Computer and Data Science Fuzhou University Fuzhou Fujian People's Republic of China; ^4^ Department of Thoracic Surgery Zhongshan Hospital (Xiamen), Fudan University Xiamen Fujian People's Republic of China; ^5^ Department of Vascular Surgery Zhongshan Hospital, Fudan University Shanghai People's Republic of China; ^6^ Department of Pulmonary and Critical Care Medicine Zhongshan Hospital, Fudan University Shanghai People's Republic of China

**Keywords:** anlotinib, lung cancer, survival

## Abstract

**Purpose:**

Anlotinib is widely used in the treatment of lung cancer. However, there remains a lack of predictive biomarkers to effectively gauge the response to anlotinib therapy. We conducted a retrospective study to preliminarily explore potential risk factors that might predict outcomes in lung cancer patients undergoing anlotinib treatment.

**Patients and Methods:**

We retrospectively analyzed lung cancer patients treated with anlotinib at our hospital between 1 June 2018 and 1 June 2021. Data were gathered from electronic medical records. Demographic and clinical characteristics of patients, progression‐free survival (PFS), and overall survival (OS) were described. Predictive factors related to treatment efficacy were preliminarily analyzed using Cox regression and Kaplan–Meier survival analyses.

**Results:**

After adjusting for potential confounders, clinical stage IV (hazard ratio [HR] = 2.52, 95% confidence interval [CI], 1.09–5.82, *p* = 0.0311), N‐terminal fragment brain natriuretic peptides (NT‐pro‐BNP) > 300 pg/mL (HR = 2.54, 95% CI, 1.17–5.52, *p* = 0.0183), and neuron‐specific enolase (NSE) > 16.3 ng/mL (HR = 1.70, 95% CI, 1.03–2.81, *p* = 0.0389) were associated with shorter OS, whereas age (HR = 0.96, 95% CI, 0.94–0.99, *p* = 0.0055) was associated with a longer PFS in fully adjusted model. Kaplan–Meier analyses of cumulative risk factors (clinical stage IV, NT‐pro‐BNP > 300 pg/mL, and NSE > 16.3 ng/mL) indicated that patients with a greater number of coexisting risk factors had significantly shorter OS (*p* < 0.0001).

**Conclusion:**

Clinical stage IV, NT‐pro‐BNP level, and NSE level were identified as independent prognostic factors for lung cancer patients undergoing anlotinib treatment. Patients with multiple high‐risk factors may derive limited benefit from anlotinib.

## Graphical abstract

Clinical stage IV, NT‐pro‐BNP level, and NSE level were independent risk factors of prognosis of lung cancer who received anlotinib treatment, and patients with multiple risk factors might have limited benefit from anlotinib.

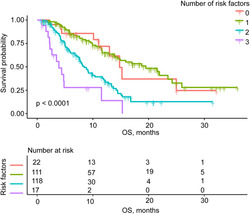

## 1 Introduction

In China, cancer remains the leading cause of deaths, with lung cancer accounting for the highest cancer‐related incidence and mortality [1]. In addition to conventional chemotherapy, newly emerging targeted therapies and immunotherapies have significantly extended the overall survival (OS) of lung cancer patients [2]. Anti‐vascular agents are considered to have satisfactory therapeutic effects, particularly in patients who are unable to tolerate chemotherapy or those with genetic mutations or pathologically proven immune‐cold tumors that are resistant to treatment [3].

Anti‐vascular therapies play a crucial role in inhibiting tumor development and metastasis. Blocking angiogenic pathways has become a widely established therapeutic strategy in cancer treatment. Anlotinib, a selective receptor tyrosine kinase inhibitor (TKI), targets vascular endothelial growth factor receptors, fibroblast growth factor receptors, platelet‐derived growth factor receptor‐α, and c‐Kit [4]. The China National Medical Products Administration has approved anlotinib for third‐line treatment of advanced non–small cell lung cancer (NSCLC) based on the results of a randomized, double‐blind, multicenter, phase III trial, ALTER‐0303 [5]. However, not all patients benefit from anlotinib, and there are currently insufficient effective predictive biomarkers to identify suitable candidates for treatment. Although substantial efforts have been made to discover potential biomarkers for anlotinib efficacy [6], many of these are not general and economical to use in clinical practice. Therefore, identifying simple and accessible prognostic biomarkers for lung cancer patients undergoing anlotinib therapy is essential. This study was conducted to preliminary investigate the risk factors influencing the prognosis of lung cancer patients treated with anlotinib.

## 2 Materials and Methods

### 2.1 Study Design and Patient Selection

**FIGURE 1:**
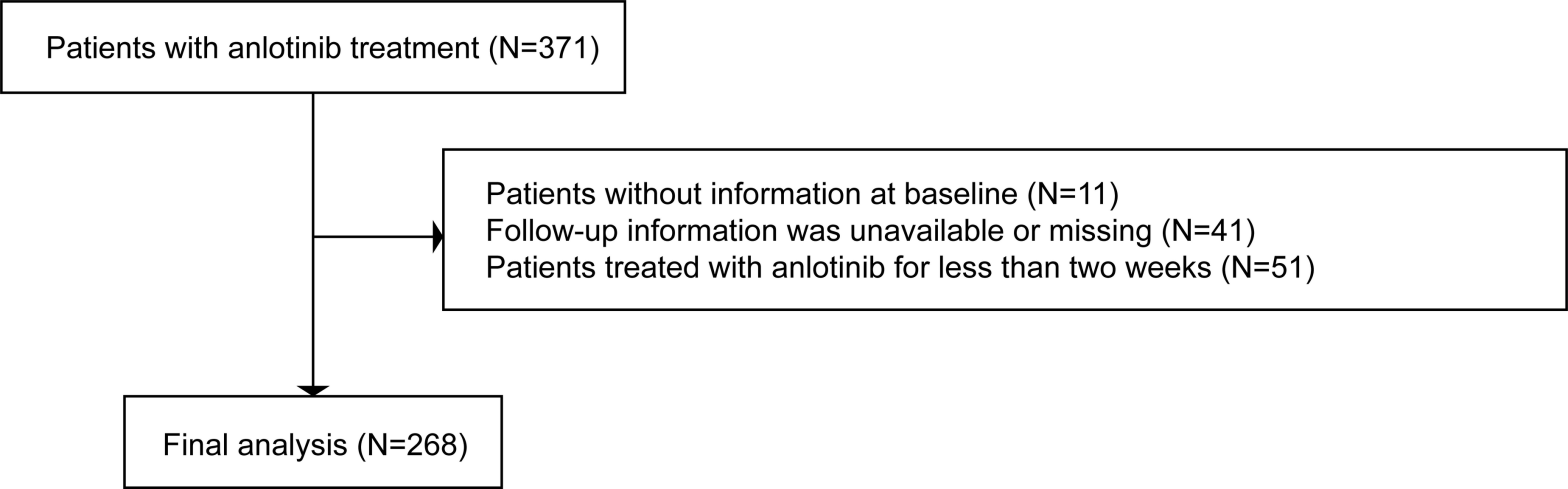
Flowchart of the study participants.

This study is an observational, retrospective, monocentric cohort study. A total of 371 lung patients, confirmed by histological or cytological examination and treated with anlotinib (either as monotherapy or in combination with one or more therapies, including chemotherapy, targeted therapy, or immunotherapy) at the Department of Pulmonary and Critical Care Medicine of Zhongshan Hospital, Fudan University, between 1 June 2018 and 1 June 2021 were consecutively enrolled initially. Anlotinib was administered at doses of 12, 10, or 8 mg daily, continuously for 2 weeks, follow by a 1‐week interruption, constituting a 3‐week cycle. After excluding 11 patients with missing baseline data, 41 patients lost to follow‐up, and 51 patients who received anlotinib treatment for less than 2 weeks, 268 patients were included in the final analysis. The research flowchart is shown in Figure 1. This study was approved by the Ethics Committee of Zhongshan Hospital (Xiamen), Fudan University (B2022‐067), and carried out following the principles of the Declaration of Helsinki. The ethics committee renounced the necessity for informed consent as this was a retrospective observational study.

### 2.2 Data Collection

Demographic data, comorbidities, clinicopathological features, genetic mutations, distant metastases, and previous treatment information were collected from electronic medical records. Baseline laboratory values were collected within 1 week prior to the first dose of anlotinib.

### 2.3 Study Assessments

The efficacy of anlotinib was assessed by the Response Evaluation Criteria in Solid Tumors version 1.1 (RECIST 1.1) base on computed tomography or magnetic resonance imaging conducted at 1.5‐month intervals or in response to clinically worsening symptoms. Progression‐free survival (PFS) was defined as the duration from the initiation of anlotinib treatment to the occurrence of tumor progression or patient death. OS was defined as the time from the start of anlotinib treatment to the date of patient death. Data collection concluded on 1 November 2021.

### 2.4 Statistical Analyses

Continuous variables are presented as means ± standards deviation (normal distribution) or median (quartiles) (skewed distribution), whereas categorical variables are presented by the frequencies and percentages. Continuous variables with skewed distribution were converted into categorical variables for subsequent analysis according to clinical reference values (Table S1). Univariate and multivariate Cox regression analyses were applied to estimate factors significantly relevant to PFS and OS. Kaplan–Meier survival analysis was utilized to appraise OS, PFS, and risk factors. All the analyses were conducted by using R statistical software (R Foundation, Vienna, Austria) and EmpowerStats (X&Y Solutions Inc., Palmetto Bay, Florida). *P* values less than 0.05 (two‐sided) were regarded as statistical significance.

## 3 Results

### 3.1 Baseline Characteristics of Participants

As shown in Figure 1, a total of 268 individuals with complete clinical, pathological, and follow‐up information were enrolled in the present research. The mean age of the patients was 61.66 ± 10.35 years, with males comprising 66.79% of the cohort. The majority of patients were diagnosed with adenocarcinoma (52.24%), followed by squamous cell carcinoma (20.52%), small cell carcinoma (17.54%), and other tissue types (9.7%). Notably, 83.58% of patients were in clinical stage IV at the time of anlotinib treatment. Additionally, 79.48% of patients received anlotinib as monotherapy, without concurrent chemotherapy, targeted therapy, or immunotherapy. Other clinical characteristics of the patients are shown in Table 1.

**TABLE 1:** Baseline characteristics of participants. Values are mean ± SD/median (Q1–Q3) or *n* (%).

Variable	Statistics
Age, years	61.66 ± 10.35
Male	179 (66.79%)
Never smoke	163 (60.82%)
Hypertension	89 (33.21%)
Histology	
Adenocarcinoma	140 (52.24%)
Squamous cell carcinoma	55 (20.52%)
Small cell carcinoma	47 (17.54%)
Others	26 (9.70%)
Clinical stage IV	224 (83.58%)
ECOG PS score > 1	26 (9.70%)
*ALK* rearrangement	
No	158 (58.96%)
Yes	3 (1.12%)
Unknown	107 (39.93%)
*EGFR* mutation	
No	94 (35.07%)
Yes	68 (25.37%)
Unknown	106 (39.55%)
Number of metastases ≥ 3	65 (24.25%)
Number of previous treatment lines ≥ 3	123 (45.90%)
Number of previous chemotherapy lines > 2	68 (25.37%)
Previous targeted therapy	81 (30.22%)
Previous radiotherapy	65 (24.25%)
Previous immunotherapy	52 (19.40%)
Anlotinib monotherapy	213 (79.48%)
Leukocyte, 10^9^/L	7.07 ± 2.86
Neutrophil, 10^9^/L	5.04 ± 2.51
Lymphocyte, 10^9^/L	1.25 ± 0.54
Platelet, 10^9^/L	249.51 ± 99.24
Albumin, g/L	41.50 ± 5.01
PT, s	11.88 ± 1.17
APTT, s	26.94 ± 3.30
Fibrinogen, mg/dL	486.07 ± 149.75
D‐dimer, mg/L	0.82 (0.40–1.70)
ALT, U/L	18.00 (13.00–24.88)
AST, U/L	22.35 (18.00–27.00)
LDH, U/L	230.00 (192.00–286.75)
BUN, mmol/L	5.82 ± 2.07
Creatinine, μmol/L	80.30 ± 23.75
NT‐pro‐BNP, pg/mL	73.20 (38.07–137.75)
CEA, ng/mL	8.80 (3.60–52.00)
NSE, ng/mL	17.15 (13.00–26.87)
CYFRA21‐1, ng/mL	5.20 (2.90–11.70)
ProGRP, pg/mL	56.90 (41.40–88.60)
SCC, ng/mL	1.40 (0.90–2.40)

Abbreviations: ALT, alanine transaminase; APTT, activated partial thromboplastin time; AST, aspartate transaminase; BUN, blood urea nitrogen; CEA, carcinoembryonic antigen; CYFRA21‐1, cytokeratin 19 fragment; ECOG PS, Eastern Cooperative Oncology Group Performance Status; LDH, lactic dehydrogenase; NSE, neuron‐specific enolase; NT‐pro‐BNP, N‐terminal fragment brain natriuretic peptides; ProGRP, progastrin‐releasing peptide; PT, prothrombin time; SCC, squamous cell carcinoma antigen.

### 3.2 Univariate Analysis

According to the univariate analysis (Table 2), factors associated with shorter OS included clinical stage IV, the ECOG PS score > 1, number of metastases ≥ 3, leukocyte, neutrophil, fibrinogen, D‐dimer, lactate dehydrogenase (LDH), NT‐pro‐BNP, neuron‐specific enolase (NSE), cytokeratin 19 fragment (CYFRA21‐1), and the squamous cell carcinoma antigen (SCC), whereas higher albumin were associated with longer OS. Adenocarcinoma, neutrophil, activated partial thromboplastin time (APTT), fibrinogen, and D‐dimer were associated with a shorter PFS, whereas age was associated with a longer PFS.

**TABLE 2:** Univariate analysis of OS and PFS.

Variable	OS	PFS
	Hazard ratio (95% CIs), *p*	Hazard ratio (95% CIs), *p*
Age, years	1.01 (1.00, 1.03), 0.0992	0.98 (0.97, 1.00), 0.0354
Female	0.90 (0.63, 1.28), 0.5563	1.09 (0.76, 1.56), 0.6287
Never smoke	0.87 (0.62, 1.23), 0.4246	0.94 (0.66, 1.35), 0.7491
Hypertension	1.00 (0.70, 1.41), 0.9828	1.04 (0.73, 1.49), 0.8334
Adenocarcinoma	0.88 (0.63, 1.22), 0.4442	1.72 (1.20, 2.45), 0.0030
Clinical stage IV	2.06 (1.22, 3.47), 0.0068	1.24 (0.77, 1.98), 0.3728
ECOG PS score > 1	2.14 (1.34, 3.41), 0.0015	1.35 (0.74, 2.45), 0.3239
*ALK* rearrangement		
No	1.0	1.0
Yes	0.87 (0.21, 3.57), 0.8483	0.36 (0.05, 2.64), 0.3155
Unknown	0.96 (0.68, 1.35), 0.8013	0.80 (0.56, 1.15), 0.2260
*EGFR* mutation		
No	1.0	1.0
Yes	1.07 (0.70, 1.64), 0.7384	0.89 (0.58, 1.38), 0.6007
Unknown	0.97 (0.66, 1.43), 0.8839	0.75 (0.50, 1.13), 0.1724
Number of metastases ≥ 3	1.65 (1.15, 2.37), 0.0072	1.07 (0.71, 1.64), 0.7368
Number of previous treatment lines ≥ 3	1.24 (0.89, 1.73), 0.1955	1.29 (0.91, 1.83), 0.1451
Number of previous chemotherapy lines > 2	1.06 (0.73, 1.54), 0.7550	1.09 (0.75, 1.59), 0.6580
Previous targeted therapy	1.26 (0.89, 1.78), 0.2020	1.40 (0.98, 2.02), 0.0665
Previous radiotherapy	0.81 (0.55, 1.20), 0.2975	0.78 (0.52, 1.17), 0.2355
Previous immunotherapy	1.11 (0.73, 1.69), 0.6280	0.84 (0.52, 1.34), 0.4532
Anlotinib monotherapy	0.93 (0.61, 1.41), 0.7247	1.07 (0.68, 1.67), 0.7773
Leukocyte, 10^9^/L	1.08 (1.02, 1.14), 0.0128	1.06 (1.00, 1.13), 0.0627
Neutrophil, 10^9^/L	1.11 (1.03, 1.18), 0.0042	1.08 (1.01, 1.17), 0.0356
Lymphocyte, 10^9^/L	0.84 (0.62, 1.15), 0.2765	0.94 (0.68, 1.30), 0.7271
Platelet, 10^9^/L	1.00 (1.00, 1.00), 0.3181	1.00 (1.00, 1.00), 0.7895
Albumin, g/L	0.93 (0.90, 0.95) < 0.0001	0.97 (0.94, 1.01), 0.1172
PT, s	1.04 (0.91, 1.18), 0.5888	1.03 (0.91, 1.17), 0.6397
APTT, s	1.01 (0.96, 1.07), 0.6333	1.07 (1.01, 1.13), 0.0217
Fibrinogen, mg/dL	1.0013 (1.0003, 1.0023), 0.0125	1.0013 (1.0002, 1.0024), 0.0200
D‐dimer > 0.8 mg/L	1.90 (1.35, 2.67), 0.0002	1.50 (1.06, 2.13), 0.0237
ALT > 50 U/L	1.42 (0.63, 3.23), 0.3985	1.99 (0.73, 5.45), 0.1810
AST > 40 U/L	1.07 (0.56, 2.03), 0.8440	0.90 (0.42, 1.93), 0.7849
LDH > 245 U/L	1.70 (1.21, 2.39), 0.0020	1.42 (0.99, 2.04), 0.0576
BUN > 8.2 mmol/L	1.15 (0.63, 2.08), 0.6503	1.20 (0.67, 2.14), 0.5326
Creatinine > 115 μmol/L	0.91 (0.48, 1.73), 0.7708	0.99 (0.53, 1.84), 0.9702
NT‐pro‐BNP > 300 pg/mL	2.50 (1.57, 3.96), 0.0001	1.19 (0.68, 2.07), 0.5493
CEA > 5 ng/mL	1.43 (0.99, 2.07), 0.0578	1.36 (0.93, 1.97), 0.1108
NSE > 16.3 ng/mL	1.93 (1.37, 2.71), 0.0002	1.19 (0.84, 1.69), 0.3337
CYFRA21‐1 > 3.3 ng/mL	1.74 (1.18, 2.55), 0.0047	1.21 (0.84, 1.75), 0.3101
ProGRP > 65.7 pg/mL	0.99 (0.70, 1.39), 0.9380	0.88 (0.61, 1.26), 0.4695
SCC > 3 ng/mL	1.55 (1.03, 2.33), 0.0351	0.64 (0.37, 1.10), 0.1042

Abbreviations: ALT, alanine transaminase; APTT, activated partial thromboplastin time; AST, aspartate transaminase; BUN, blood urea nitrogen; CEA, carcinoembryonic antigen; CI, confidence interval; CYFRA21‐1, cytokeratin 19 fragment; ECOG PS, Eastern Cooperative Oncology Group Performance Status; LDH, lactic dehydrogenase; NSE, neuron‐specific enolase; NT‐pro‐BNP, N‐terminal fragment brain natriuretic peptides; ProGRP, progastrin‐releasing peptide; PT, prothrombin time; SCC, squamous cell carcinoma antigen.

### 3.3 Multivariate Analysis

The multivariate analysis of OS is presented in Table 3, showing both the minimally adjusted model and the fully adjusted model. Compared with the univariate analysis, minimally adjusted model (only adjusted for age and gender) showed clinical stage IV, ECOG PS score > 1, number of metastases ≥ 3, leukocyte, neutrophil, LDH, fibrinogen, D‐dimer, NT‐pro‐BNP, NSE, CYFRA21‐1, and SCC were still associated with shorter OS, whereas albumin was associated with longer OS. However, in the fully adjusted model, only clinical stage IV (HR = 2.52, 95% CI, 1.09–5.82, *p* = 0.0311), NT‐pro‐BNP > 300 pg/mL (HR = 2.54, 95% CI, 1.17–5.52, *p* = 0.0183), and NSE > 16.3 ng/mL (HR = 1.70, 95% CI, 1.03–2.81, *p* = 0.0389) remained associated with shorter OS. The multivariate analysis of PFS (Table 4) indicated that age was associated with longer PFS in minimally adjusted model (only adjust for gender), whereas adenocarcinoma, neutrophil, APTT, fibrinogen, and D‐dimer were associated with shorter PFS. However, only age (HR = 0.96, 95% CI, 0.94–0.99, *p* = 0.0055) remained associated with a longer PFS after fully adjusted.

**TABLE 3:** Multivariate analysis of OS.

Variable	Minimally adjusted model	Fully adjusted model
	Hazard ratio (95% CIs), *p*	Hazard ratio (95% CIs), *p*
Clinical stage IV	2.21 (1.30, 3.75), 0.0034	2.52 (1.09, 5.82), 0.0311
ECOG PS score > 1	2.01 (1.25, 3.25), 0.0041	1.37 (0.59, 3.19), 0.4705
Number of metastases ≥ 3	1.69 (1.17, 2.43), 0.0049	1.34 (0.80, 2.26), 0.2681
Leukocyte, 10^9^/L	1.07 (1.01, 1.14), 0.0253	0.93 (0.45, 1.92), 0.8456
Neutrophil, 10^9^/L	1.10 (1.02, 1.18), 0.0085	1.21 (0.56, 2.63), 0.6307
Albumin, g/L	0.93 (0.90, 0.96) < 0.0001	0.93 (0.75, 1.16), 0.5395
ALP > 125 U/L	1.92 (1.22, 3.01), 0.0045	1.44 (0.71, 2.92), 0.3132
LDH > 245 U/L	1.76 (1.25, 2.47), 0.0011	1.29 (0.79, 2.10), 0.3151
Fibrinogen, mg/dL	1.001 (1.000, 1.002), 0.01979	1.001 (0.999, 1.003), 0.16586
D‐dimer > 0.8 mg/L	1.85 (1.31, 2.62), 0.0004	1.43 (0.86, 2.37), 0.1639
NT‐pro‐BNP > 300 pg/mL	2.43 (1.49, 3.98), 0.0004	2.54 (1.17, 5.52), 0.0183
NSE > 16.3 ng/mL	1.93 (1.37, 2.71), 0.0002	1.70 (1.03, 2.81), 0.0389
CYFRA21‐1 > 3.3 ng/mL	1.73 (1.18, 2.54), 0.0051	1.29 (0.75, 2.20), 0.3557
SCC > 3 ng/mL	1.52 (1.00, 2.31), 0.0483	1.47 (0.84, 2.58), 0.1770

*Note:* Minimally adjusted model adjust for age and gender. Fully adjusted model adjust for age; gender; never smoke; hypertension; histology; clinical stage; ECOG PS score; *ALK* rearrangement; *EGFR* mutation; number of metastases; number of previous treatment lines; number of previous chemotherapy lines; previous targeted therapy; previous radiotherapy; previous immunotherapy; anlotinib monotherapy; leukocytes; neutrophil; lymphocyte; platelets; albumin; PT; APTT; fibrinogen; D‐dimer; ALT; AST; LDH; BUN; creatinine; NT‐pro‐BNP; ProGRP; CEA; NSE; CYFRA21‐1; and SCC, excepting for the stratified variable itself.

**TABLE 4:** Multivariate analysis of PFS.

Variable	Minimally adjusted model	Fully adjusted model
	Hazard ratio (95% CIs), *p*	Hazard ratio (95% CIs), *p*
Age, years	0.98 (0.97, 1.00), 0.0407	0.96 (0.94, 0.99), 0.0055
Adenocarcinoma	1.74 (1.18, 2.55), 0.0048	1.83 (0.73, 4.61), 0.1968
Neutrophil, 10^9^/L	1.10 (1.02, 1.19), 0.0094	0.69 (0.29, 1.63), 0.4011
APTT, s	1.07 (1.01, 1.14), 0.0143	1.06 (0.96, 1.17), 0.2488
Fibrinogen, mg/dL	1.00 (1.00, 1.00), 0.0064	1.00 (1.00, 1.00), 0.3283
D‐dimer > 0.8 mg/L	1.59 (1.12, 2.27), 0.0102	1.53 (0.85, 2.73), 0.1548

*Note:* Minimally adjusted model adjust for age and gender, excepting for the stratified variable itself. Fully adjusted model adjust for age; gender; never smoke; hypertension; histology; clinical stage; ECOG PS score; *ALK* rearrangement; *EGFR* mutation; number of metastases; number of previous treatment lines; number of previous chemotherapy lines; previous targeted therapy; previous radiotherapy; previous immunotherapy; anlotinib monotherapy; leukocytes; neutrophil; lymphocyte; platelets; albumin; PT; APTT; fibrinogen; D‐dimer; ALT; AST; LDH; BUN; creatinine; NT‐pro‐BNP; ProGRP; CEA; NSE; CYFRA21‐1; and SCC, excepting for the stratified variable itself.

### 3.4 Kaplan–Meier Analyses

**FIGURE 2:**
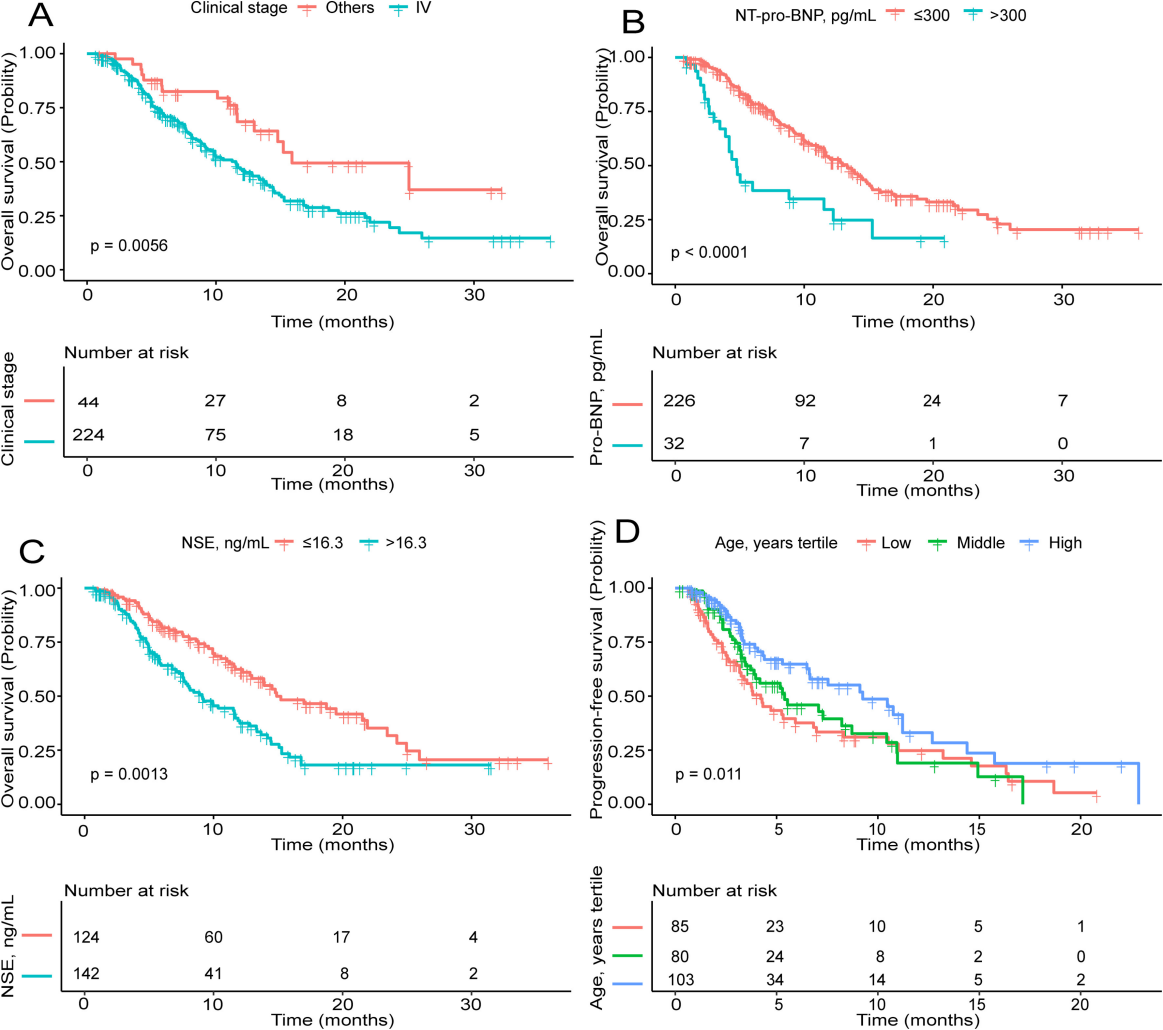
(A) Kaplan–Meier survival curves for OS were compared among different clinical stage groups. (B) Kaplan–Meier survival curves for OS were compared among different NT‐pro‐BNP groups. (C) Kaplan–Meier survival curves for OS were compared among different NSE groups. (D) Kaplan–Meier survival curves for PFS were compared among different age groups.

**FIGURE 3:**
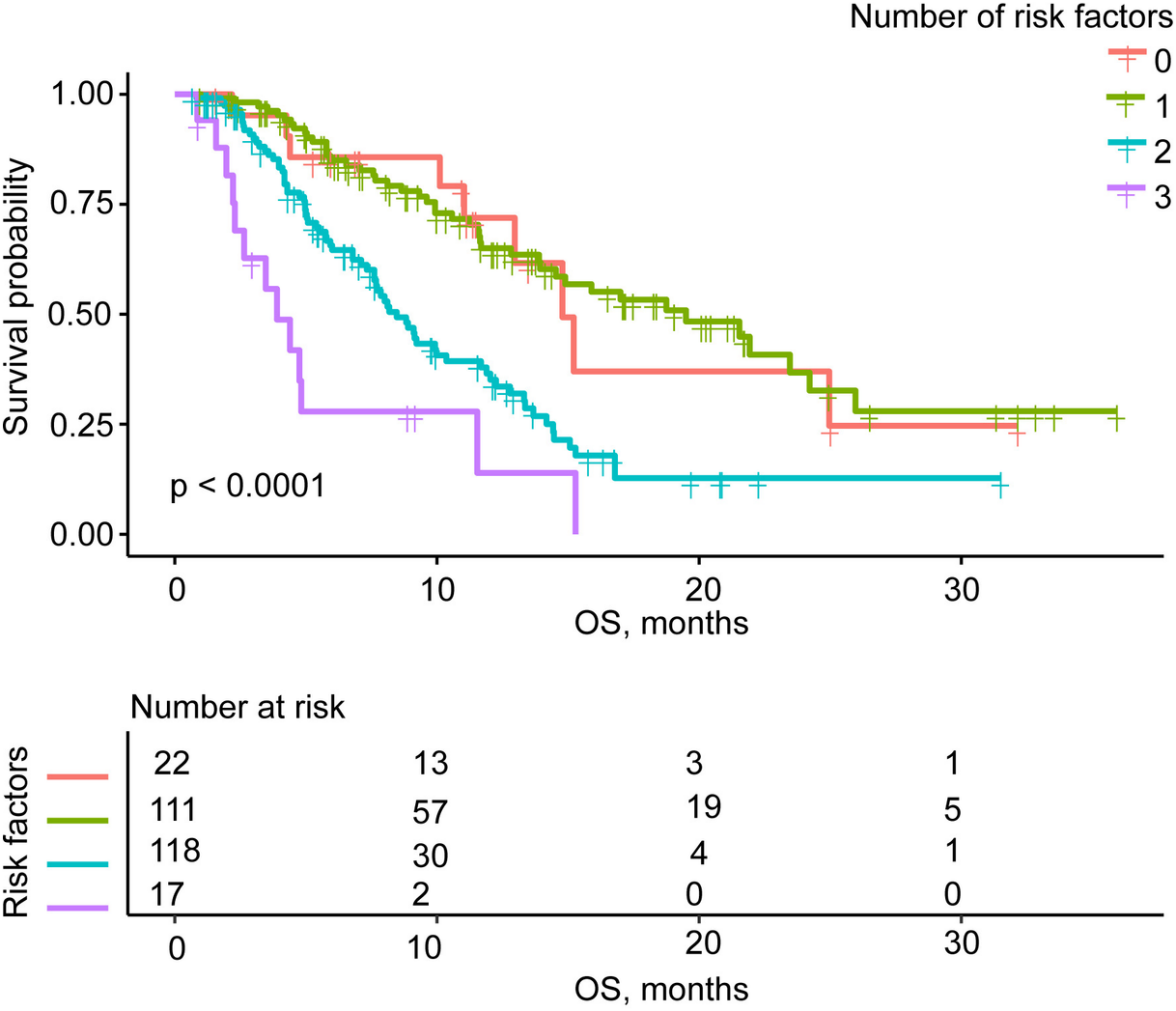
Kaplan–Meier survival curves for OS were compared among different number of risk factors groups.

Kaplan–Meier analyses demonstrated that clinical stage IV (*p* = 0.0056), NT‐pro‐BNP > 300 pg/mL (*p* < 0.0001), and NSE > 16.3 ng/mL (*p* = 0.0013) were associated with worse OS, whereas older patients demonstrated longer PFS (*p* = 0.011) (Figure 2A–D). According to the results of multivariate analysis of OS (fully adjusted model), the number of risk factors was recorded, including clinical stage IV, NT‐pro‐BNP > 300 pg/mL, and NSE > 16.3 ng/mL. Kaplan–Meier analyses of OS and the number of risk factors indicated that patients with a greater number of risk factors were associated with shorter OS (*p* < 0.0001) (Figure 3).

## 4 Discussion

Anlotinib is a novel antiangiogenic drug, which serves an essential role in alleviating the proliferation and invasion of malignant tumor cells. Previous studies have confirmed that anlotinib has promising anticancer effectiveness and safeness in both SCLC and NSCLC patients [7, 8]. However, the criteria for inclusion in clinical trials are rigorous. Studies in which to investigate anlotinib treatment in a real‐world setting are finite. We retrospectively appraised data from lung cancer patients treated with anlotinib to assess factors influencing efficacy and to determine therapeutically dominant populations who could have more clinical benefit from anlotinib.

Univariate analysis identified 14 factors affecting OS and 6 factors affecting PFS, primarily related to patient characteristics and baseline laboratory value (Table 2). No therapeutic factors were found to have a significant impact on OS or PFS. Based on these findings, the identified parameters were introduced into the multivariate Cox model. In the fully adjusted model, clinical stage IV, baseline NT‐pro‐BNP, and NSE increase were significantly associated with shorter OS (Table 3), whereas older age was associated with longer PFS (Table 4). These results suggest that clinical stage IV, NT‐pro‐BNP, and NSE were independent risk factors of OS. Furthermore, Kaplan–Meier analyses indicated that an increased number of risk factors, including clinical stage IV, NT‐pro‐BNP > 300 pg/mL, and NSE > 16.3 ng/mL, were associated with shorter OS (Figure 3).

In terms of patient characteristics, previous studies have suggested that age serves as a predictive indicator of anlotinib treatment efficacy in NSCLC patients treated with anlotinib combined with immunotherapy [9]. The mPFS of elderly patients was shorter than that of younger patients. In contrast, the mOS for individuals aged 70–75 years undergoing anlotinib as third‐line or later treatment was 14.5 months, which exceeded the mOS of 7.9 months for those aged 60–70 years and 10.1 months for patients under 60 years [5]. These findings indicated that elderly patients, particularly those over 75 years, may benefit from anlotinib therapy. In our study, age emerged as an independent protective factor for PFS in lung cancer patients treated with anlotinib, with a small hazard ratio (just 0.96), though it showed no significant association with OS. Given the controversial role of age in different studies, further large prospective cohort studies are warranted.

Additionally, clinical stage emerged as a significant independent prognostic factor in our study. Patients at clinical stage IV had markedly shorter OS compared to those in earlier stages. This finding is intuitive, as a later clinical stage means the tumor has likely been growing for a longer period, providing more opportunities for spread and invasion to other organs. Therefore, early screening is necessary for high‐risk lung cancer patients, as those diagnosed at an earlier stage may derive greater benefit from anlotinib treatment.

Previous studies have shown that patients with EGFR‐sensitive mutations may be an optimal population for antiangiogenic therapies [10, 11]. However, in our study, there was no significant difference in mPFS and mOS based on whether the EGFR status was mutated, wild type, or unknown. These findings are consistent with the ALTER0303 study, which also demonstrated that the therapeutic effect of anlotinib on advanced NSCLC is independent of EGFR status.

Current studies have shown that higher ECOG PS scores are associated with poorer prognoses in patients with malignant tumor [12, 13, 14], and ECOG PS may serve as an independent factor for PFS [15, 16]. One possible explanation for these findings is that the score data in retrospective studies were documented poorly and tardy compared to that in clinical trials. Besides, hypertension status has been identified as an independent factor influencing PFS in patients with extensive‐stage SCLC receiving anlotinib monotherapy [15]. In our study, there was no significant difference in PFS and OS between groups based on hypertension status, suggesting that hypertension may not be a contraindication for anlotinib use, despite the fact that anlotinib can lead to Grade 3–4 hypertension in up to 13.6% of patients [7]. Nevertheless, the conclusions of our study require validation through larger‐scale prospective trials.

Regarding treatment strategies, previous studies have shown that combining anlotinib with other therapies can enhance the objective response rate (ORR) and disease control rate (DCR) in SCLC patients, resulting in marginally prolonged PFS and OS [17]. In another study, anlotinib combined with chemotherapy was associated with extended PFS and OS in second‐line treatment of SCLC patients compared to anlotinib alone, although there was no significant improvement in mPFS or mOS in third‐line and later treatments [18]. In our study, anlotinib monotherapy appeared to prolong PFS while shortening OS, although these differences did not reach statistical significance.

It has traditionally been believed that increasing lines of chemotherapy can induce multidrug resistance (MDR) in tumor cells [19], resulting in tolerance to various chemotherapeutic agents. Consequently, it is well recognized that second‐line regimens are often less effective than first‐line treatments. Similarly, EGFR‐TKIs can lead to T790M mutations and *c‐Met* gene amplification, contributing to resistance to first‐generation drugs [20, 21]. However, in our study, the number and types of prior treatments, which have been associated with the development of tumor resistance in previous reports, did no show a statistically significant effect on prognosis following anlotinib treatment.

Thoracic radiotherapy is a well‐established intervention aimed at stabilizing local thoracic tumors. Subgroup analyses of ALTER0303 and ALTER1202 studies have shown that NSCLC and SCLC patients receiving anlotinib therapy exhibited longer PFS if they had previously undergone radiotherapy [22, 23]. This enhancement may be attributed to the alteration of the immune microenvironment induced by thoracic radiotherapy, which could potentially increase the efficacy of anlotinib [22]. However, in our study, both univariate and multivariate analyses revealed no significant association between previous radiotherapy and OS or PFS in lung cancer patients receiving anlotinib treatment. This lack of association may be due to the small sample size of our study. Therefore, prospective, large‐scale cohort studies are warranted to elucidate the effect of chest radiotherapy on the prognosis of lung cancer patients treated with anlotinib.

Some routine clinical laboratory test results can serve as prognostic factors for lung cancer patients receiving anlotinib treatment, offering a convenient and noninvasive means for survival prediction. Our findings indicated that elevated levels of NT‐pro‐BNP and NSE were associated with worse OS, and multivariate analysis confirmed that NT‐pro‐BNP and NSE levels could serve as independent markers for OS. NT‐pro‐BNP is secreted by cardiomyocytes during the stretching of the atrial or ventricular walls, playing an important role in remodeling, volume homeostasis, and ischemic response [24]. Previous studies have established NT‐pro‐BNP as an important diagnostic tool and predictor in acute coronary syndromes and heart failure [25, 26]. Furthermore, elevated NT‐pro‐BNP levels have been linked to cardiac metastases in lung cancer patients [27].

Anlotinib, as a multitargeted TKIs, can disrupt normal tyrosine kinase function in non‐neoplastic cells, leading to various toxicities, including cardiotoxic effects such as hypertension, congestive heart failure, and cardiac ischemia [28]. A meta‐analysis of angiogenesis inhibitors showed a nearly threefold increased risk of cardiac ischemia and a 1.26‐fold greater risk of fatal cardiovascular events [29]. Hence, we hypothesized that anlotinib may adversely affect OS through its cardiotoxicity, potentially exacerbating issues in lung cancer patients with existing cardiomyocyte abnormalities. Further studies are required to confirm this hypothesis, and our findings underscore the importance of evaluating cardiac function when prescribing anlotinib for lung cancer.

NSE, a cytoplasmic enzyme primarily expressed in neurons and neuroendocrine cells, serves as a specific serum marker of neuronal damage, [30]. It is elevated in cancers of neuroendocrine cellular origin, including SCLC, with a specificity about 85%. NSE can be used for survival prognosis, treatment monitoring, and recurrence prediction [31, 32]. The role of NSE as a predictive and prognostic marker in NSCLC remains controversial. Tiseo et al. reported that higher baseline serum NSE levels in advanced NSCLC were associated with response to standard first‐line chemotherapy but did not establish a prognostic role [33]. Fiala et al. showed that high baseline NSE levels did not predict outcomes in NSCLC patients treated with EGFR‐TKIs [34]. However, our study showed that the patients with elevated baseline NSE levels faced a 1.7‐fold increased risk of worse OS compared to those with normal NSE levels, suggesting that NSE may hold prognostic value in lung cancer patients receiving anlotinib therapy. Moreover, we innovatively performed Kaplan–Meier analyses on the three independent risk factors for OS identified in this study, revealing that a higher number of coexisting risk factors was associated with shorter OS in lung cancer patients treated with anlotinib. This finding may provide a theoretical basis for screening superior lung cancer patients for anlotinib treatment in the future.

This study has several limitations. First, the patient data were sourced from a single medical institution, and the sample size was relatively small. Consequently, this may introduce bias, highlighting the need for larger‐scale observational studies to validate these findings. Additionally, we were unable to investigate adverse reactions comprehensively, as the date collected were not based on case report forms (CRFs) and lack of consistent monitoring. Third, the limited number of targeted cases led to relatively broad inclusion criteria. Consequently, future studies should aim to stratify analyses based on clinical stages, particularly for Stage II, III, and IV patients, to provide clearer insights into the predictive effects of identified biomarkers. Finally, as more cases become available in the future, we plan to conduct specific analyses for various subtypes.

In conclusion, our study identified clinical stage IV, NT‐pro‐BNP levels, and NSE levels as independent risk factors for OS in lung cancer patients undergoing anlotinib treatment. Additionally, age emerged as an independent protective factor for PFS. Notably, a higher number of coexisting risk factors were associated with shorter OS.

## Author Contributions

Congyi Xie conducted the partial data analyses and wrote the initial manuscript. Jinzhan Chen and Shuwen Yang contributed to data collection. Feiyang Ye conducted partial data analyses. Zhenyang Lin, Yijiao Xu, and Yimin Yang participated in the revision of the manuscript and the follow‐up of partial data. Lin Tong revised the data analyses and the manuscript. All authors read and approved the final manuscript.

## Ethics Statement

The present study has been approved by the Ethics Committee of Iran University of Zhongshan Hospital (Xiamen), Fudan University (B2022‐067).

## Conflicts of Interest

The authors declare no conflicts of interest.

## Supporting information


**Table S1** Reference value of clinical laboratory test results.

## Data Availability

The data that support the findings of this study are available from the corresponding author upon reasonable request.
