# Supplementary material for: Risk Factors for Prognosis of Lung Cancer Patients Receiving Anlotinib Treatment: A Retrospective Cohort Study
Source: Clin Respir J. 2025 Feb 9;19(2):e70051. doi: 10.1111/crj.70051 (PMC11807704; doi:10.1111/crj.70051)
Supplement: Supplementary file 1 — Table S1 Reference value of clinical laboratory test results. [file CRJ-19-e70051-s001.doc]

**Supplement Table 1 Reference value of clinical laboratory test results.**

| Variable | Clinical reference |
| --- | --- |
| Leukocyte, 10^9/L | 3.50-9.50 |
| Neutrophil, 10^9/L | 1.8-6.3 |
| Lymphocyte, 10^9/L | 1.1-3.2 |
| Platelet, 10^9/L | 125-350 |
| Albumin, g/L | 35-55 |
| PT, sec | 10.0-13.0 |
| APTT, sec | 25.0-32.7 |
| Fibrinogen, mg/dL | 185-400 |
| D-dimer, mg/L | 0-0.80 |
| ALT, U/L | 9-50 |
| AST, U/L | 15-40 |
| LDH, U/L | 109-245 |
| BUN, mmol/L | 2.9-8.2 |
| Creatinine, μmol/L | 44-115 |
| NT-pro-BNP, pg/mL | 0-300 |
| CEA, ng/mL | <5 |
| NSE, ng/mL | <16.3 |
| CYFRA21-1, ng/mL | <3.3 |
| ProGRP, pg/mL | <65.7 |
| SCC, ng/mL | <3 |
